# Cognitive bias modification training of attention and interpretation to reduce expectations of social rejection in adolescents with eating disorders: A small efficacy randomized controlled trial

**DOI:** 10.1002/eat.23809

**Published:** 2022-09-22

**Authors:** Katie Rowlands, Taryn Beaty, Mima Simic, Ben Grafton, Colette Hirsch, Janet Treasure, Valentina Cardi

**Affiliations:** ^1^ Department of Psychological Medicine Institute of Psychiatry, Psychology and Neuroscience, King's College London London UK; ^2^ Child and Adolescent Eating Disorders Service South London and Maudsley NHS Foundation Trust London UK; ^3^ Centre for the Advancement of Research on Emotion School of Psychological Science, University of Western Australia Crawley Australia; ^4^ Psychology Department Institute of Psychiatry, Psychology and Neuroscience, King's College London London UK; ^5^ Department of General Psychology University of Padova Italy

**Keywords:** anorexia nervosa, attention, bias, interpretation, online, social rejection

## Abstract

**Objective:**

This study aimed to investigate whether a computerized cognitive bias modification training delivered remotely would reduce expectations of rejection in adolescents with eating disorders.

**Method:**

Sixty‐seven adolescents aged 12–18 (99.5% female) with an eating disorder diagnosis (94% anorexia nervosa) and receiving specialist treatment were recruited. Participants were randomized to an intervention condition (*n* = 37) which included treatment as usual (TAU) supplemented by nine sessions of online cognitive bias modification training for social stimuli (CBMT + TAU), or a control condition (*n* = 30), which included TAU only. Participants were invited to complete assessments at baseline and post‐intervention.

**Results:**

In the intervention condition, 22/37 participants completed six or more training sessions and post‐intervention measures, the pre‐defined criteria to be considered “completers.” In the control condition, 28/30 participants completed the post‐intervention measures. Participants who completed the intervention displayed a significantly greater reduction in negative interpretations of ambiguous social scenarios, with a medium effect size (*p* = .048, ηp2 = .090), and eating disorder psychopathology, with a medium effect size (*p* = .027, ηp2 = .105), compared to participants in the control condition. No significant between‐group differences were found on emotional response to criticism, and anxiety and depression symptoms post‐intervention (*p*s > .05; small effect sizes).

**Discussion:**

Enhancing treatment as usual with CBMT targeting expectations of social rejection might be feasible and effective to reduce expectations of social rejection and eating disorder psychopathology in adolescents with eating disorders. Training adaptations might be necessary to impact on emotional processing and comorbid psychological distress.

**Public Significance:**

Adolescents with eating disorders who completed a brief (4‐week) online cognitive training intervention, alongside their usual treatment, reported greater reductions in expectations of social rejection and eating disorder psychopathology after the intervention, compared to a separate group of patients who received their usual treatment only. This brief and accessible intervention may be a helpful treatment adjunct for adolescents with eating disorders.

## 1 INTRODUCTION

Eating disorders are serious mental illnesses that are typically triggered during adolescence, an important stage in the lifespan characterized by profound physical, psychological and social development (Rapee et al., 2019). While these conditions are diagnostically characterized by problems with eating, weight and shape, interpersonal difficulties are prevalent too. Due to the increased salience of peer relationships and heightened sensitivity to rejection during adolescence (Lam et al., 2014; Somerville, 2013), the impact of negative interpersonal experiences is likely to be most influential to the development and maintenance of eating disorders at this time.

### 1.1 Social difficulties in eating disorders

In many cases, people with anorexia nervosa recall fewer social interactions and difficulties interacting with peers from an early age (Krug et al., 2012; Westwood et al., 2016). Some longitudinal data suggest that social difficulties in childhood can play a predisposing role in the development of eating disorders, predicting clinical cases at age 14 (Allen et al., 2009). This proposal has been supported by patients themselves, who have recognized the role that social difficulties played in the development of their illness (Cardi, Mallorqui‐Bague, et al., 2018). Specific interpersonal traits, such as shyness, submissiveness, and an increased tendency toward social comparison are known risk factors (Treasure & Schmidt, 2013). Additional interpersonal problems have been identified in the acute state of the illness (Cardi, Tchanturia, & Treasure, 2018). The secondary effects of starvation and chronic stress have a detrimental impact on social cognition (Caglar‐Nazali et al., 2014) and contribute to social isolation (Treasure et al., 2020). Furthermore, the instinctive interpersonal reactions to the illness from close others can inadvertently perpetuate the symptoms (Schmidt & Treasure, 2006; Treasure et al., 2020; Treasure & Schmidt, 2013).

Adolescents with anorexia nervosa report submissive behaviors, unfavorable social comparison, sensitivity to rejection and poorer quality of social relationships to a greater extent than their healthy peers (Cardi et al., 2019; Kalaycı et al., 2019; Rowlands et al., 2021). Some of these problems, for example greater alienation perceived within family and peer relationships (Pelletier Brochu et al., 2018) and heightened interpersonal sensitivity (Rowlands et al., 2021), have been associated with a greater severity of self‐reported eating disorder psychopathology. In qualitative studies, adolescents with eating disorders have described a poor sense of group belonging, a heightened tendency to engage in self‐monitoring, and rejection sensitivity (Patel et al., 2016). They have also referred to premorbid and illness‐related problems within the social context outside of the family (Lindstedt et al., 2018).

### 1.2 Cognitive biases toward negative social stimuli in adolescents

Cognitive biases toward negative social stimuli have been identified as modifiable mechanisms underlying interpersonal difficulties in adolescents with a diagnosis of mental disorders, as well as adolescents with heightened symptoms of psychological distress (predominantly anxiety or depression; Platt et al., 2017; Stuijfzand et al., 2018). The most broadly investigated cognitive biases in adolescents include selective patterns of attention toward negative or disorder‐related stimuli (attention bias; Puliafico & Kendall, 2006; Roy et al., 2015) and a tendency to interpret ambiguous social information in a negative or threatening way (interpretation bias; Platt et al., 2017; Stuijfzand et al., 2018).

### 1.3 Cognitive biases toward negative social stimuli in eating disorders

Given that people with eating disorders are highly sensitive to social feedback, studies have begun to investigate the presence of these cognitive biases and their association with core eating disorder psychopathology, as well as anxiety and depression, in this population. Most of these studies have been conducted in adults. For example, one study found that a mixed sample of adults with anorexia nervosa or bulimia nervosa demonstrated attention biases toward rejecting facial expressions, which were associated with adverse childhood experiences (Cardi et al., 2013). Another study demonstrated that adults with anorexia nervosa made more negative interpretations of ambiguous social scenarios that involved a risk of rejection compared to healthy controls, and that this bias was associated with fear of weight gain, body image disturbance and anxiety and depression symptoms (Cardi et al., 2017). Negative interpretation biases have also been found in adolescents with eating disorders and are associated with eating disorder psychopathology (Cardi et al., 2019; Rowlands et al., 2021).

### 1.4 Cognitive bias modification training for social stimuli in adolescents

Cognitive bias modification training (CBMT) procedures have been designed to reduce attention biases toward negative social stimuli (Bar‐Haim, 2010) and negative interpretations of ambiguous social information (Hirsch et al., 2009). These trainings have been predominantly tested in adolescents with anxiety disorders or depression, or in adolescents with heightened anxiety or depression symptoms (Gober et al., 2021). There is a growing evidence base for the efficacy and effectiveness of these procedures at modifying near‐transfer outcomes (e.g., cognitive biases), and far‐transfer outcomes, such as clinical symptoms, in clinical samples of adolescents (Hang et al., 2021; Lau et al., 2021;Salemink et al., 2015; Wolters et al., 2021). For example, adolescents with anxiety disorders who received attention bias modification in addition to cognitive behavioral therapy (CBT) demonstrated a greater reduction in anxiety symptoms post‐intervention compared to the control group, who received CBT only (Riemann et al., 2013). Furthermore, adolescents with depression who received an active attention bias modification training (training attention away from sad words and toward neutral and positive words) demonstrated greater reductions in attention bias and depressive symptoms compared to a control group, who received a placebo attention bias modification training. These findings were maintained at 1‐year follow‐up (Yang et al., 2016). Studies using interpretation bias training for anxiety in adolescents have focused on non‐clinical samples, however two studies showed that adolescents with depression who received interpretation bias training demonstrated small to moderate reductions in symptoms and small differences in post‐intervention scores compared to the control group (LeMoult et al., 2018; Micco et al., 2014). More recently, some preliminary data have shown that cognitive bias modification training can reduce obsessive–compulsive interpretations and clinical symptoms in adolescents with obsessive–compulsive disorder (Salemink et al., 2015; Wolters et al., 2021).

### 1.5 Cognitive bias modification training for social stimuli in eating disorders

Cognitive bias modification procedures have been shown to reduce attention biases toward negative social stimuli (facial expressions) in adults with anorexia nervosa, and negative interpretation biases of ambiguous social scenarios in both adults and adolescents with anorexia nervosa (Cardi et al., 2015; Cardi et al., 2019; Turton et al., 2018). Two of these studies have investigated the impact of the training on response to social rejection in this population. In one of these studies, adults with anorexia nervosa reported lower anxiety and higher self‐compassion in response to a judgemental video clip (Cardi et al., 2015). In the second study, adolescent patients demonstrated a trend toward higher self‐esteem in response to virtual social exclusion induced by the Cyberball paradigm (Cardi et al., 2019).

Given the value of tailoring interpretation bias stimuli to the population of interest (Hughes et al., 2016) we have adapted the interpretation bias training for adults with eating disorders, with the goal of making the scenarios included in the training more relevant for adolescents. This process involved conducting focus groups with patients, carers and professionals, developing a set of scenarios based on real experiences of rejection among adolescents with eating disorders, and collecting quantitative feedback from patients on the relevance and emotional salience of the scenarios (Rowlands et al., 2020).

The current study examines the efficacy of multi‐session CBMT, including attention bias modules and interpretation bias modification modules, with content developmentally tailored to, and produced in collaboration with, adolescents with eating disorders. The aim of the study was to pilot‐test the effects of adding a multi‐session CBMT to treatment as usual (TAU) for adolescents with eating disorders, in comparison to TAU only. Based on the available evidence for the effects of CBMT in adolescents with anxiety or depression (Lau et al., 2021), we hypothesized that participants who completed CBMT alongside TAU would report greater reductions in negative interpretation biases of ambiguous social scenarios and attention biases toward social rejection‐related stimuli (rejecting faces) compared to participants who received TAU only. We also hypothesized that participants who completed would report greater reductions in eating disorder psychopathology, lower emotional response to criticism, and greater reductions in depression, and anxiety symptoms, compared to participants who received TAU only.

## 2 METHOD

### 2.1 Design

Sixty‐seven adolescents with eating disorders receiving clinical care, including outpatient, day care or inpatient care were randomly allocated to one of two conditions: (1) online CBMT + TAU, or (2) TAU only. Participants were randomized using random numbers generated on an excel spreadsheet, with 50% probability of being allocated to either condition. Self‐report measures and computerized tasks were administered at baseline and post‐intervention. All outcomes were assessed at post‐intervention. The main outcome measures were negative interpretation bias, as measured by negative “best” completions produced on the sentence completion task, and changes in negative attention bias, as measures by attention bias scores toward critical faces in the attentional probe assessment task. Secondary outcomes included self‐reported eating disorder psychopathology, depression and anxiety symptoms, and emotional response to criticism, as measured by a single item visual analogue scale in response to a critical video.

Participants allocated to the intervention condition were invited to complete nine sessions of CBMT in addition to their clinical care, over 4 weeks, and assessment measures at baseline and post‐intervention. Due to the lack of studies that have evaluated multi‐session protocols in clinical samples of adolescents (Krebs et al., 2018), the number and frequency of CBMT sessions required to have the desired effects in participants are currently uncertain. In this study, to qualify as “completers” participants were required to complete at least six out of the nine sessions of the training. This design was guided by similar multi‐session studies in clinical samples of adults with anxiety or depression which demonstrated therapeutic effects on clinical symptoms (Amir & Taylor, 2012; Blackwell & Holmes, 2010; Lam et al., 2014; Williams et al., 2015). Studies in these fields were consulted in particular, as they have a longer history of piloting multi‐session CBMT protocols (Gober et al., 2021) compared to clinical samples of adolescents (Krebs et al., 2018).

Participants allocated to the control conditon received treatment as usual and were invited to complete the study assessment measures at baseline and post‐intervention. After participants in the control condition had completed the study, they received the link to the training sessions.

Treatment as usual included either outpatient, day care or inpatient treatment. Due to the additional pressures of the Covid‐19 pandemic on clinical teams, we did not request specific details about participants' individual treatment from clinical services. Typically, outpatient care for adolescents with eating disorders includes weekly or less than weekly appointments with a specialist eating disorder professional for physical health checks, and psychological treatment such as family therapy / individual therapy. Treatment for patients receiving day or inpatient treatment typically involves daily or almost‐daily support from a multi‐disciplinary team. This includes a more intensive regime of regular physical health checks, a range of therapies (i.e., family, individual, group therapy), and support to improve nutrition and weight (National Institute for Health and Care Excellence, 2017). Due to the Covid‐19 lockdown, some treatment was delivered more flexibly (e.g., online).

### 2.2 Participants

Adolescents receiving eating disorder treatment were invited to participate. Participants were recruited from two specialist child and adolescent eating disorder services in London and from the community via online advertisements and social media. Inclusion criteria were aged 12–18, fluency in English; with an eating disorder diagnosis, and receiving psychological treatment. Exclusion criteria were severe comorbid psychiatric disorders (e.g., psychosis), severe visual or hearing impairment, or neurological disorders. Eating disorder diagnoses were self‐reported by patients.

We based our power calculation on interpretation bias change because negative interpretation biases have been found in adolescents with eating disorders but not attention biases toward critical faces. Prior research using a multi‐session protocol of attention and interpretation bias training sessions in people with eating disorders demonstrated a small to medium reduction in negative interpretations on the sentence completion task (Cardi et al., 2015). We used G*Power to calculate the sample size based on detecting a medium effect size (*f* = .25) achieving 80% power, at *p* = .05, using repeated‐measures analyses of variance. Based on this calculation, a minimum of 42 participants in total would be needed. We aimed to recruit a minimum of 60 participants to account for potential drop‐out (20%–30%).

### 2.3 Measures

#### 2.3.1 Demographic and clinical characteristics questionnaire

At baseline, participants completed a brief demographic and clinical questionnaire which included questions to assess the following variables: age, gender, ethnicity, weight, height, duration of eating disorder, and comorbid psychiatric diagnoses.

#### 2.3.2 Eating Disorder Examination Questionnaire (EDE‐Q; Fairburn & Beglin, 1994)

The 36‐item, self‐report “Eating Disorder Examination Questionnaire” (EDE‐Q; Fairburn & Beglin, 1994), was used to measure core eating disorder psychopathology at baseline and post‐intervention. The measure consists of four subscales (dietary restraint, eating concerns, weight concerns and shape concerns). Participants rated the frequency of experiencing these problems in the past 28 days, on a scale ranging from *no days* (0) to *every day* (6). A score on each subscale and a total score is calculated. Reliability was high for the total score (*α* = .95), and subscales for restraint (*α* = .84), eating concerns (*α* = .79), weight concerns (*α* = .84) and shape concerns (*α* = .92).

#### 2.3.3 Revised Children's Anxiety and Depression Scale‐25 (RCADS‐25; Chorpita et al., 2000)

The short (25‐item) self‐report “Revised Children's Anxiety and Depression Scale‐25” (RCADS‐25; Chorpita et al., 2000) was used to measure symptoms of anxiety and depression in the past 28 days at baseline and post‐intervention. The scale consists of two subscales (anxiety symptoms, and depression symptoms). Participants rated the frequency of experiencing these symptoms over the past 28 days, on a scale ranging from *never* (0) to *always* (3). A score on each subscale and a total score is calculated. Reliability was high for the total score (*α* = .92) and anxiety (*α* = .84) and depression (*α* = .89) subscales.

#### 2.3.4 Interpersonal Sensitivity Measure (IPSM; Boyce & Parker, 1989)

The 36‐item, self‐report “Interpersonal Sensitivity Measure” (IPSM; Boyce & Parker, 1989) was used as a measure of sensitivity to rejection at baseline. The measure consists of five subscales including interpersonal awareness, need for approval, separation anxiety, timidity, and fragile inner self. Participants rate the extent to which each item is true for them on a four‐point Likert scale ranging from 1 (“very unlike me”) to 4 (“very like me”). A score for each subscale and a total score was calculated. Reliability was good for the total score (*α* = .89), timidity (*α* = .81), fragile inner‐self (*α* = .82), separation anxiety (*α* = .83) and interpersonal awareness (*α* = .76), but lower for need for approval (*α* = .57). These alpha values were adequate and comparable to those found in previous studies, including the original validation study involving adults (Boyce & Parker, 1989) and in adolescents with depressive spectrum disorders (Masillo et al., 2014).

#### 2.3.5 Attention bias: Attentional probe assessment task (MacLeod et al., 1986)

An adapted version of the attentional probe assessment task (MacLeod et al., 1986) was administered to assess attention toward positive and negative social stimuli (rejecting and accepting faces) at baseline and post‐intervention. The face image stimuli were derived from the Max Planck database (Ebner et al., 2010) and were used to create two face subsets, which we refer to as face subset A (128 face pairs) and face subset B (128 face pairs). For a full description of the task, see Supplementary Materials Item 1.

#### 2.3.6 Interpretation bias: Sentence completion task (Huppert et al., 2007)

An adapted version of the sentence completion task (Huppert et al., 2007) was used to measure the valence of interpretations of ambiguous social scenarios (either as positive/neutral, or negative) at baseline and post‐intervention. The scenarios were adapted from a previous study in adolescents with eating disorders (Cardi et al., 2019) by the researchers, to increase the relevance to the age group included in this study. In each trial, participants read on the computer screen, and listened to with headphones, a stem sentence describing an ambiguous social scenario, and were asked to enter as many completions to the given scenario as they could in 90 seconds. Participants then indicated the word completion which would “best” complete the sentence using an asterisk. Participants listened to two practice trials followed by ten test trials. The first ten completions made for the ten test trials were rated by two members of the research team (K.R., T.B.) who were blinded to condition allocation. Each completion was rated as neutral/positive or negative. The number of completions were capped at 10 for each scenario. In line with previous research reporting three indexes of interpretation bias (Cardi et al., 2017), six raw scores per stem were calculated for each participant. These included a score for the total number of negative completions and a score for the total number of positive completions (out of 100 possible completions across the ten stems). For the total negative or positive completions only, mean scores were calculated to account for variance in the number of completions made (participants varied in the number of completions they made for each stem). The number of negative and positive “first” completions were scored using the ratings of the first completion that the participants made on each of the ten stems, and the number of negative and positive “best” completions were scored using the sum of negative or positive ratings of the completions that participants selected as “best” completing the scenario across the ten stems.

#### 2.3.7 Social rejection video clips (Chami et al., personal communication)

The video clips was developed by researchers in the eating disorders lab at King's College London (Chami et al., personal communication). The video clips and Likert scales were administered at post‐intervention only and were used to measure emotional response to criticism in the CBMT + TAU and TAU groups. Participants were presented with video clips of actors making six statements in total, split into three pairs of two statements. Participants were asked to rate how hurt they felt after watching the actors make these two statements on a Likert scale, ranging from 1 (not at all hurt) to 10 (extremely hurt). Two neutral statements “There are sixty minutes in one hour,” and “New Mexico is in the United States” were followed by two critical statements, “Your laugh annoys me,” “Do you always look so run down?,” and two further critical statements “Everyone thinks you look so weird,” and “No one is drawn to you.”  

#### 2.3.8 Cognitive bias modification of attention (CBM‐A): Attentional probe training task (Cardi et al., 2013)

An adapted version of the attentional probe assessment task (Cardi et al., 2013) was used to modify attention toward positive or neutral social stimuli (faces). Each of the training sessions included 128 trials, which were split across two blocks of 64 trials. Each session began with the presentation of a fixation point (+) for 500 ms. The inter‐trial interval was 500 ms. The same face identities used in the baseline assessment task (Ebner et al., 2010) were used in the training tasks (i.e., participants who completed the assessment with faces from subset A completed the training using the same faces from subset A). Each face identity was presented once, before being presented again. The first time the face was shown it was either presented showing a critical or happy expression, and the second time it was shown it was presented showing the opposite expression. There were an equal number of trials on which a male versus female face was presented, and an equal number of trials on which the emotional face appeared on the left versus right‐hand side of the screen. However, unlike the assessment task, in the training task the probe (a faint gray line which is either horizontally or vertically orientated) always appeared (100% of the time) in the location opposite to the rejecting face, and in the same location as the smiling face. Participants were instructed to respond immediately to the presentation of the probe, by pressing the corresponding letter on the keyboard (i.e., the “V” or “H” key).

#### 2.3.9 Cognitive bias modification of interpretation (CBM‐I): Ambiguous scenarios training task (Hirsch et al., 2009)

An adapted version of the ambiguous scenarios training task (Hirsch et al., 2009) was used to train benign or neutral interpretations of ambiguous social scenarios. The scenarios included in the training were developed based on feedback from young people with eating disorders, carers and professionals (see: Rowlands et al., 2020 for details on the process of stimuli development). In each session, participants were presented with 50 of the hypothetical social scenarios (as text on the screen and via audio clips using headphones). The scenarios were ambiguous, in that they depicted the possibility of rejection. On “modification” trials, the scenarios were resolved after a few seconds with a resolution which is either positive or negative in valence. In this study, there were 44 modification trials. On 38 of these trials, the scenarios were completed with a benign ending (76%). Six of the scenarios were completed with a negative ending (12%). Six “catch” trials (12%) were also included, which retained their ambiguity (i.e., they were not resolved in an explicit positive or negative way). Participants were encouraged to complete a total of nine training sessions. In all trials, following the presentation of each scenario, participants were asked a “comprehension question” which they had to answer “yes” or “no” to. In modification trials, the answer to this comprehension question was followed by feedback (for 1000 ms) indicating whether the answer was correct or by a sound indicating that the answer was incorrect, followed by the correct answer highlighted in red text. On catch trials, no feedback was provided. The catch trials were presented at random points in between the modification trials. The wording of scenarios and comprehension questions were balanced, so that the “correct” answer was “yes” or “no” with equal contingencies, which could reinforce either a positive or negative interpretation (see Table S1 for an example of each trial type). Participants were encouraged to complete a total of nine CBM‐I training sessions. Following recommendation from previous studies (Hertel & Mathews, 2011; Mathews & Mackintosh, 2000), a small number of negative trials were included, to ensure that participants were attending carefully to the content of the scenarios included in the training, and to provide intermittent reinforcement which more accurately reflects real life. Catch trials were included as a supplementary indicator of interpretation bias change during the task.

**FIGURE 1:**
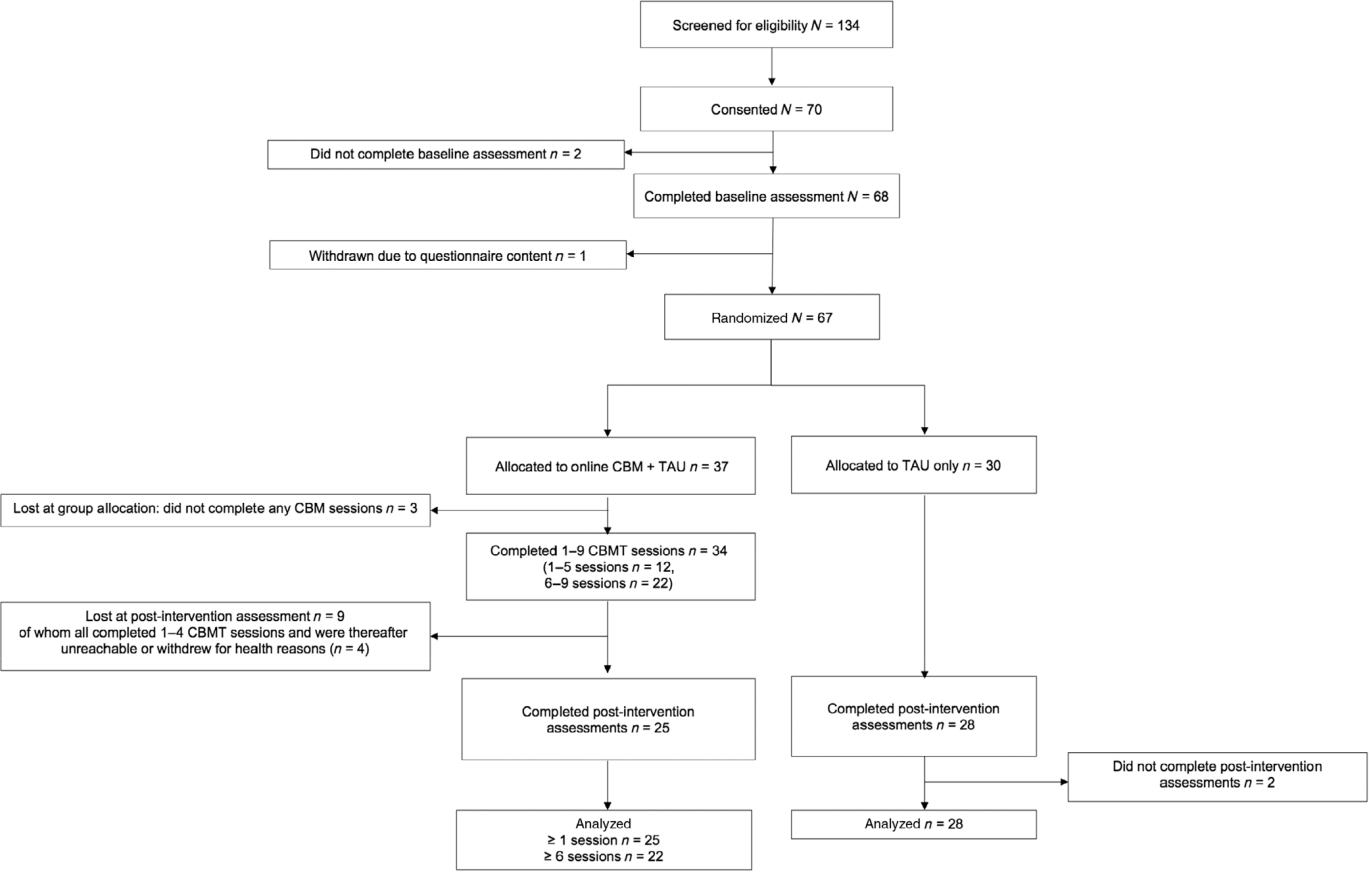
Consort diagram of participants in the study, including the cognitive bias modification training + treatment as usual (TAU) and the TAU groups. The flow chart describes participant recruitment and completion of assessments at baseline and post‐intervention, and the completion of cognitive bias modification training (CBMT) sessions in the intervention group.

#### 2.3.10 Feedback form

At post‐intervention, participants in the CBMT + TAU group completed a brief feedback form including open questions about the intervention (see Table S4 for the questions asked and a summary of participant responses).

### 2.4 Procedure

The study was conducted during the Covid‐19 pandemic, from April 2020 until June 2021. This study involved human participants and was reviewed and approved by London—Riverside Research Ethics Committee (REC Ref: 17/LO/1867). According to our ethically approved protocol, participants aged below 16 years of age provided assent to participate in this study. Parental consent was also obtained. For a full description of study procedure, see Supplementary Materials Item 2.

### 2.5 Data analyses

Data were analyzed using SPSS Version 26. Missing data were not imputed for this study, thus missing data reduced the sample sizes in the analyses. Assumptions of normality were explored using the Shapiro Wilk Test. Independent Samples T‐Tests were used to compare baseline demographic and clinical characteristics between the two groups. To prepare the attention bias assessment data at baseline and post‐intervention for analysis, inaccurate trials were excluded from participants' total score (in cases where, for example, participants selected the “V” for vertical key on the keyboard when a horizontal line appeared). Trials were not excluded based on reaction times. Participants were excluded if they reported overall accuracy below 80%. Repeated measures ANOVAs were used to examine changes between groups over time in attention and interpretation biases and clinical symptoms (eating disorder psychopathology, anxiety and depression symptoms). Effect sizes were calculated using Cohen's *d* and partial eta squared. Due to violation of normality, a non‐parametric test (Mann–Whitney *U*) was used to compare the two groups on emotional response to the critical video clips.

## 3 RESULTS

Sixty‐seven adolescents who were receiving specialist eating disorder treatment (*n* = 67) were recruited. As shown in Figure 1, 37 participants were randomly allocated to the intervention condition (CBMT + TAU), and 30 were allocated to the control condition (TAU only). In the intervention group, three participants never started the training, and 12 participants completed 1–5 training sessions. Twenty‐two participants completed a minimum of six sessions (out of nine available sessions) and were considered “completers.” The majority of participants who completed the intervention did so within 4 weeks, with five participants (23%) exceeding this (range = 3–16 weeks). Twenty‐five participants in the CBMT + TAU group completed the post‐intervention measures. In the control group, post‐intervention measures were completed by 28 of 30 participants. The majority of participants who completed the post‐intervention measures (*n* = 41, 77%) did so within 4 weeks, with 12 participants exceeding this (range 3–24 weeks). Participants in the intervention condition who did not complete the training scored higher on the EDE‐Q global score, *t*(36) = −3.128, *p* = .003. This finding suggests that participants who had more severe eating disorder symptoms struggled to engage in the study. Figure 1 describes participant recruitment, and rates of adherence to the training and completion of assessments. The results which follow refer to the comparison between the intervention completers and controls. The results from the intention‐to‐treat analysis are also reported for participants in the CBMT + TAU group who completed at least one session of the training, and the post‐intervention measures (*n* = 25) compared to controls (see Tables S2 and S3).

### 3.1 Baseline demographic and clinical characteristics

In the overall sample (*N* = 67), participants reported high levels of interpersonal sensitivity, as indicated by the global score of the interpersonal sensitivity measure (*M* = 112.80, *SD* = 12.54). In a previous study, adolescents with eating disorders scored significantly higher on this measure compared to healthy controls (Rowlands et al., 2021). Participants scored highly on the severity of eating disorder psychopathology, based on norms for adolescents with anorexia nervosa (Jennings & Phillips, 2017), and 40/67 (60%) scored in the clinical range for the EDE‐Q total, based on the widely used cut‐off score of 4 (Meule, 2021). On average, the overall sample scored highly on depression symptom severity (*M* = 71.40, *SD* = 16.79) with 38/67 (57%) participants scoring in the clinical range (≥70). The sample scored relatively lower on anxiety symptoms (*M* = 63.67, *SD* = 13.85) with 20/67 (30%) scoring in the clinical range (≥70).

Baseline demographic and clinical characteristics for participants included in the main analyses are presented in Table 1. Participants in the intervention group reported a longer duration of illness compared to the control group. Additionally, significantly more participants in the intervention group reported depression or anxiety as a comorbidity.

Hypothesis 1 *Participants who completed CBMT alongside TAU would report greater reductions in negative interpretation biases of ambiguous social scenarios and attention biases toward social rejection‐related stimuli* (*rejecting faces*) *compared to participants who received TAU only*.

**TABLE 1:** Demographic and clinical characteristics of participants in the cognitive bias modification training + treatment as usual (CBMT + TAU) group completers and TAU groups

	*N* (%) or *M* (*SD*)				
Baseline characteristics	All *N* = 50	CBMT + TAU (*n* = 22)	TAU (*n* = 28)	Test statistic	Effect size
Age	15.85 (1.74)	16.36 (1.59)	15.47 (1.78)	*t*(50) = 1.879, *p* = .066	*d* = .6
Gender				*p* = .423	*φ* = .164
Girls	49 (98)	21 (95)	30 (100)		
Boys	1 (2)	1 (5)	0 (0)		
Ethnicity				*p* = .502	*φ* = .212
White British	48 (96)	22 (100)	26 (92.9)		
Asian	1 (2)	0 (0)	1 (3.6)		
Other	1 (2)	0 (0)	1 (3.6)		
Eating disorder diagnosis				*p* = .360	*φ* = .284
Anorexia nervosa	46 (92)	20 (91)	26 (92.9)		
Bulimia nervosa	2 (4)	2 (9)	0 (0)		
Avoidant restrictive food intake disorder	1 (2)	0 (0)	1 (3.6)		
Eating disorder not otherwise specified	1 (2)	0 (0)	1 (3.6)		
Duration of eating disorder (months)	18.08 (21.72)	27.09 (26.18)	11.86 (15.48)	*t*(32) = 2.417 *p* = .021*	*d* = .70
Comorbidities				*p* = .048*	*φ* = .300
Depression	15 (3)	10 (45)	5 (18)		
Bipolar disorder	1 (2)	0 (0)	1 (3.5)		
Anxiety disorder	13 (26)	9 (41)	4 (14)		
Autism spectrum disorder	1 (2)	1 (4.5)	0 (0)		
Obsessive compulsive disorder	1 (2)	1 (4.5)	0 (0)		
Multiple comorbidities	10	8 (36)	2 (7)		
Treatment				*p* = .793	*φ* = .164
Outpatient	44 (88)	19 (86)	25 (89)		
Day care	5 (10)	3 (14)	2 (7)		
Inpatient	1 (2)	0 (0)	1 (4)		
Psychiatric medication	23 (46)	8 (36)	15 (54)	*p =* .403	*φ* = −.136
Weight for height percentile	22.28 (22.99)	20.68 (25.94)	21.41 (21.11)	*t*(44) = −.392, *p* = .697	*d* = .12
IPSM interpersonal awareness	24.62 (3.27)	24.81 (3.25)	24.46 (3.44)	*t*(48) = .370, *p* = .713	*d* = .10
IPSM need for approval	25.79 (2.94)	26.18 (2.89)	25.64 (2.90)	*t*(48) = .654, *p* = .516	*d* = .19
IPSM separation anxiety	24.37 (4.67)	24.68 (5.12)	24.07 (4.54)	*t*(48) = .446, *p* = .657	*d* = .13
IPSM timidity	23.50 (4.27)	23.68 (3.50)	23.79 (4.65)	*t*(48) = −.087, *p* = .931	*d* = .03
IPSM fragile inner self	13.67 (3.75)	14.18 (3.61)	13.21 (3.99)	*t*(48) = .887, *p* = .380	*d* = .25
IPSM total	111.94 (13.04)	113.55 (12.63)	111.18 (13.77)	*t*(48) = −.597, *p* = .553	*d* = .18
EDE‐Q eating restraint	3.25 (1.75)	3.18 (1.83)	3.20 (1.73)	*t*(50) = −.239, *p* = .812	*d* = .01
EDE‐Q eating concerns	3.21 (1.45)	3.09 (1.55)	3.23 (1.40)	*t*(50) = −.511, *p* = .612	*d* = .09
EDE‐Q weight concerns	3.88 (1.61)	3.59 (1.50)	4.03 (1.71)	*t*(50) = −1.114, *p* = .270	*d* = .27
EDE‐Q shape concerns	4.57 (1.45)	4.32 (1.32)	4.71 (1.58)	*t*(50) = −1.040, *p* = .303	*d* = .27
EDE‐Q total	3.73 (1.42)	3.55 (1.41)	2.98 (1.37)	*t*(50) = −.780, *p* = .439	*d* = .41
RCADS‐25 depression	69.84 (17.90)	69.74 (19.49)	69.38 (17.29)	*t*(50) = −.033, *p* = .974	*d* = .02
RCADS‐25 anxiety	63.29 (15.21)	66.37 (17.34)	60.92 (13.73)	*t*(50) = 1.257, *p* = .214	*d* = .35
RCADS‐25 total	68.29 (17.49)	70.47 (20.15)	66.38 (15.83)	*t*(50) = .765, *p* = .448	*d* = .23

*Note*: This table displays demographic and clinical characteristics collected from participants at baseline. The table presents data for the sample included in the analysis overall (*n* = 50) and for the two study groups including the cognitive bias modification training + treatment as usual (CBMT + TAU) group “completers” and the TAU group. Fisher's exact test was used to compare groups on gender, ethnicity, eating disorder diagnosis, comorbidities, treatment type and medication status. Data presented as means (*M*), standard deviations (*SD*) or counts with percentages, test statistics, and significance values. *d* = Cohen's *d* measure of effect size.

Abbreviations: EDE‐Q, Eating Disorder Examination Questionnaire; IPSM, Interpersonal Sensitivity Measure; RCADS‐25, Revised Child Anxiety and Depression Scale—Short Form.

**FIGURE 2:**
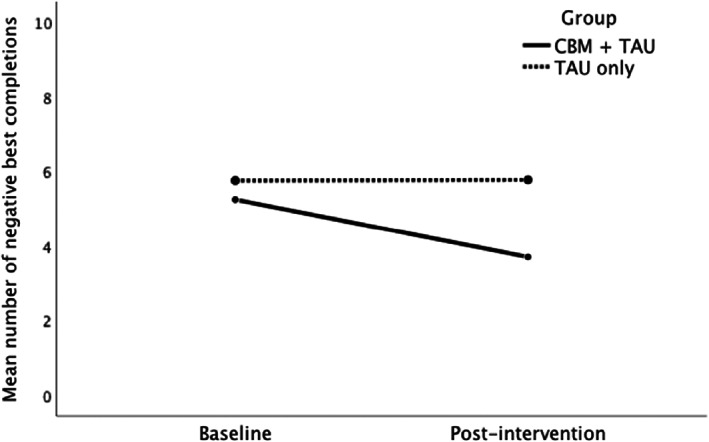
Mean negative “best” completions in the sentence completion task for the cognitive bias modification training + treatment as usual (CBMT + TAU) group completers and treatment as usual (TAU) group at baseline and post‐intervention. This graph illustrates the mean number of negative “best” completions of ambiguous social scenarios in the sentence completion task in the CBMT + TAU group completers (*n* = 22) and TAU group (*n* = 28) at baseline and post‐intervention. Covariates appearing in the model are evaluated at the following values: eating disorder duration (months) = 18.02, depression = 1.70, anxiety = 1.74.

As shown in Table 2, there was a significant Time x Group interaction effect on negative interpretation bias. Specifically, there was a significantly greater reduction in negative “best” completions produced on the sentence completion task from baseline to post‐intervention in the CBMT + TAU group compared to the TAU group, when controlling for duration of illness, depression, and anxiety disorder comorbidities (medium effect size; see Figure 2). There was also a greater trend‐level reduction in negative “first” completions in the CBMT + TAU group compared to the TAU group (medium effect size). However, there was no significant Time × Group interaction effect for total negative completions. There was also no significant reduction in attention bias toward rejecting faces.

Hypothesis 2 *Participants who completed CBMT alongside TAU would report a greater reduction in eating disorder symptoms, emotional response to criticism*, *depression*, *and anxiety symptoms compared to participants who received TAU only*.

**TABLE 2:** Cognitive biases, clinical measures and emotional response to criticism at baseline and post‐intervention in the cognitive bias modification training + treatment as usual (CBMT + TAU) group completers and TAU groups

	Baseline *M* (*SD*)	Post‐intervention *M* (*SD*)	Mean difference M [95% CI]	Baseline *M* (*SD*)	Post‐intervention *M* (*SD*)	Mean difference *M* (95% CI)	
Measure	CBMT + TAU *n* = 21			TAU *n* = 26			Test statistics
Total negative completions	25.48 (12.42)	18.62 (13.03)	−6.86 [−11.76 to −1.96]	28.27 (14.68)	23.58 (10.57)	−4.69 [−10.33 to .94]	Time *F*(1, 45) = 9.712, *p* = .003*, ηp2 = .178 Group *F*(1, 45) = 1.418, *p* = .240, ηp2 = .031 Time × Group *F*(1, 45) = .341, *p* = .562, ηp2 = .008
First negative completions	5.86 (1.28)	4.24 (1.81)	−1.62 [−2.53 to −.70]	6.54 (2.60)	6.12 (1.84)	−.42 [−1.39 to .55]	Time *F*(1, 45) = 9.676, *p* = .003*, ηp2 = .177 Group *F*(1, 45) = 7.164, *p* = .010*, ηp2 = .137 Time × Group *F*(1, 45) = 3.319, *p* = .075, ηp2 = .069
Best negative completions	5.57 (1.91)	3.76 (2.12)	−1.81 [−2.61 to −1.01]	5.50 (3.02)	5.73 (2.13)	.23 [−.78 to 1.24]	Time *F*(1, 42) = .301, *p* = .586, ηp2 = .007 Group *F*(1, 42) = 3.362, *p* = .074, ηp2 = .074 Time × Group *F*(1, 42) = 4.135, *p* = .048*, ηp2 = .090 Time × Depression *F*(1, 42) = .352, *p* = .556, ηp2 = .008 Time × Anxiety *F*(1, 42) = .084, *p* = .773, ηp2 = .002 Time × Duration of eating disorder *F*(1, 42) = 1.383, *p* = .246, ηp2 = .032
Attention bias to critical faces	−14.85 (110.27)	−87.42 (434.80)	−72.57 [−274.16 to 129.01]	−35.94 (97.93)	−42.58 (164.56)	−6.64 [−79.84 to 66.56]	Time *F*(1, 44) = .674, *p* = .416, ηp2 = .015 Group *F*(1, 44) = .055, *p* = 815, ηp2 = .001 Time × Group *F*(1, 44) = .467, *p* = .498, ηp2 = .010
EDE‐Q total	3.55 (1.41)	2.97 (1.37)	−5.75 [.97 to .18]	3.79 (1.46)	3.64 (1.47)	−.15 [.33 to .04]	Time *F*(1, 45) = 2.068, *p* = .157, ηp2 = .044 Group *F*(1, 45) = 8.278, *p* = .006*, ηp2 = .155 Time × Group *F*(1, 45) = 5.261, *p* = .027*, ηp2 = .105 Time × Depression *F*(45) = 1.796, *p* = .187, ηp2 = .038 Time × Anxiety *F*(1, 45) = .676, *p* = .415, ηp2 = .015 Time × Duration of eating disorder *F*(1, 45) = 1.948, *p* = .170, ηp2 = .042
RCADS‐25 anxiety	66.37 (17.34)	64.12 (17.98)	−2.25 [−7.93–3.43]	60.92 (13.73)	57.15 (15.33)	−3.77 [−8.50 to .97]	Time *F*(1, 48) = 2.867, *p* = .097, ηp2 = .056 Group *F*(1, 48) = 2.193, *p* = .145, ηp2 = .044 Time × Group *F*(1, 44) = .182, *p* = .672, ηp2 = .004
RCADS‐25 depression	69.74 (19.49)	66.93 (17.08)	−2.81 [−8.71 to 3.08]	69.38 (17.29)	66.12 (17.33)	−3.26 [−8.45 to 1.92]	Time *F*(1, 48) = 2.558, *p* = .116, ηp2 = .051 Group *F*(1, 48) = .016, *p* = .901, ηp2 = .000 Time × Group *F*(1, 48) = .014, *p* = .907, ηp2 = .000
RCADS‐25 total	70.47 (20.15)	67.62 (19.52)	−2.85 [−9.33 to 3.63]	66.38 (15.83)	62.35 (16.84)	−4.03 [−9.31 to 1.25]	Time *F*(1, 48) = 2.950, *p* = .092, ηp2 = .058 Group *F*(1, 48) = .987, *p* = .326, ηp2 = .020 Time × Group *F*(1, 48) = .087, *p* = .770, ηp2 = .002
Emotional response to criticism		11.88 (5.38)			14.04 (4.04)		*U* = 176, *p* = .262

*Note*: This table presents data for the main study variables collected from participants at baseline and post‐intervention. Analyses of negative “best” completions and EDE‐Q total include depression, anxiety and eating disorder duration as covariates. Emotional response to criticism = combined score on visual analogue scale after presentation of video sets 1 and 2. Data presented as means (*M*), standard deviations (*SD*), mean difference, 95% confidence intervals, test statistics, significance values, and ηp2 = partial eta squared (effect sizes). *p < .05.

Abbreviations: EDE‐Q, Eating Disorder Examination Questionnaire; RCADS‐25, Revised Children's Anxiety and Depression Scale—Short Form.

As shown in Table 2, there was a significant Time × Group interaction effect on eating disorder psychopathology, as indicated by the total EDE‐Q score, from baseline to post‐intervention, when controlling for duration of illness, depression and anxiety disorder as comorbidities (medium effect size). There were no significant between‐group differences in emotional response to the critical videos at post‐intervention, or depression and anxiety symptoms from baseline to post‐intervention (small effect size).

## 4 DISCUSSION

The aim of this small efficacy study was to examine the effects of adding online CBMT to treatment as usual in adolescents with eating disorders. The study hypotheses were partially supported. Patients who received CBMT + TAU showed a significantly greater decrease in negative interpretations of ambiguous social scenarios and eating disorder psychopathology compared to the TAU group. However, they did not report a significantly greater reduction in attention biases toward rejecting faces, lower emotional response to criticism post‐intervention, or significant reductions in anxiety or depression symptoms.

The finding that the CBMT + TAU group demonstrated greater reductions in negative interpretation biases post‐training is consistent with an earlier feasibility study in adolescents with anorexia nervosa, which demonstrated a decrease in negative interpretation biases following a single‐session interpretation bias modification training (Cardi et al., 2019). This finding is also consistent with evidence for decreases in negative interpretation biases following cognitive bias modification training in other clinical samples of adolescents with depression (LeMoult et al., 2018; Micco et al., 2014) and in those with obsessive–compulsive disorder (Salemink et al., 2015; Wolters et al., 2021).

In contrast to adults with eating disorders (Cardi et al., 2015), adolescents who received CBMT + TAU in this study did not demonstrate significant changes in attention biases toward rejecting faces. One explanation is that the effectiveness of attention bias modification training may depend on baseline attention bias (Price et al., 2016 ). In this study, a Wilcoxon signed‐rank test showed that 11/22 (50%) participants in the intervention group demonstrated an attention bias toward rejecting faces before the training and that this reduced to 6/22 (27%) who demonstrated this bias after completing the training. This reduction was significant (*p* = .025). This reduction provides a positive indication that the training may help to reduce attention biases toward rejecting faces in those who display a bias at baseline. However, this preliminary finding requires replication. Furthermore, due to the limitations of the attention bias paradigm used in this study, for example inadequate test–retest reliability (MacLeod et al., 2019) it is not possible to conclude that adolescents with eating disorders do not have this attention bias based on their performance on this occasion. More sensitive measures of attention bias, such as eye‐tracking, may produce different results (Kerr‐Gaffney et al., 2019). It is also possible that alternative attention bias modification paradigms may be more effective at modifying attention biases in adolescents with eating disorders, such as visual search protocols (Hang et al., 2021) gamified procedures (Notebaert et al., 2018) or virtual reality‐based attention bias modification tasks (Urech et al., 2015).

The reduction in eating disorder psychopathology in participants who completed the CBM training is encouraging, given that the training protocol was developed following recommendations for producing symptom change. These considerations included the use of multiple training sessions (Hallion & Ruscio, 2011; Hang et al., 2021), and the development of interpretation bias module materials for the specific population of interest (Hughes et al., 2016; Rowlands et al., 2020). These findings are consistent with studies using multiple sessions of interpretation bias training to modify interpretation biases in clinical samples of adolescents with depression (LeMoult et al., 2018; Micco et al., 2014) and preliminary evidence in youth with obsessive–compulsive disorder (Wolters et al., 2021). These findings also add to the existing evidence demonstrating that cognitive bias modification trainings can improve far transfer outcomes in clinical samples, such as greater reductions in anxiety when added to cognitive behavioral therapy for adolescents with anxiety disorders (Riemann et al., 2013) and fewer obsessive–compulsive symptoms in adolescents with obsessive–compulsive disorder (Salemink et al., 2015).

The failure to find a reduction in emotional sensitivity to the critical video post‐intervention, and no change in anxiety and depression symptoms may suggest that alternative measures of emotional response to a stressful interpersonal situation may be more sensitive to change. For example, adults with anorexia nervosa showed reduced anxiety and increased self‐compassion in response to a judgemental video clip was seen after five sessions of CBMT (Cardi et al., 2015). Another study found a trend for higher self‐esteem in response to the Cyberball task in adolescent patients after one experimental training session of CBM‐I (Cardi et al., 2019). These findings may also suggest that the training itself requires further adaptations to have far‐transfer effects. For example, interpretation bias modules could include content that focuses more on emotional processing of perceived rejection, in addition to encouraging more positive interpretations of ambiguity in relation to social interactions.

### 4.1 Strengths

A strength of the study was that the stimuli for the interpretation bias training modules were developed in collaboration with people with eating disorders, carers and professionals. This has rarely been the case in previous studies in this population (Rowlands et al., 2020). It is likely that the rigorous process undergone to develop the scenarios for the interpretation bias training played a role in reducing negative interpretations and eating disorder symptoms in the participants in this study, emphasizing the need to develop CBM‐I trainings in collaboration with the population of interest (Hughes et al., 2016; Lau et al., 2021).

To our knowledge this is the first study to examine the effects of a multi‐session combined CBMT protocol, using a randomized controlled trial design, in a clinical sample of adolescents with eating disorders. Our findings suggest that the training did have a positive impact on post‐intervention measures of interpretation bias and eating disorder symptoms.

### 4.2 Limitations

While this study was appropriately powered for the analysis, there are limitations to acknowledge in relation to the sample. First, the sample was relatively small and consisted of predominantly white adolescent girls, with anorexia nervosa, receiving outpatient care, who were recruited at different stages of their illness and treatment. It is not possible to conclude that these findings would be generalizable to all young people with eating disorders. Furthermore, 15/37 (41%) participants allocated to the intervention group did not complete the training. While we collected feedback from completers (see Table S4), we did not collect feedback from non‐completers. It is important that future studies capture this information in order to better understand the facilitators and barriers to engagement with CBMT.

Second, the participant recruitment and delivery of the intervention took place at the very beginning of the Covid‐19 pandemic lockdown and was conducted entirely online. At this time, to reduce burden on clinical teams, we did not routinely ask for clinician‐recorded information as long as participants were able to self‐report key information (e.g., eating disorder diagnosis, treatment intensity). We did not collect detailed information on individual participants' treatment as usual. Finally, the intervention and control groups were not matched on demographic or clinical characteristics due to randomization.

An additional limitation of this study is that the findings presented here are based on pre‐post intervention assessments, with the intervention group completing both the attention and interpretation bias tasks as part of the training. It is possible that the interpretation bias training modules could have trained participants to respond “correctly” on the measure related to that which they were trained in (the sentence completion task). Additionally, these data do not allow us to conclude whether or not the changes in interpretation bias and eating disorder psychopathology in the intervention group are maintained over time, and whether these changes are related to a critical component of the intervention (i.e., interpretation bias training or attention bias training). Nonetheless, the change in interpretation bias but not attention bias in those who completed the combined CBMT gives a positive indication that interpretation bias training is likely to have played an important role. Counterbalancing these two types of cognitive bias modification trainings (CBM‐I and CBM‐A) would be useful in future research to help delineate the contributions of each training method.

Given that this is the first study to examine the effects of a multi‐session CBMT protocol in a clinical sample of adolescents with eating disorders, the optimal CBMT protocol that could produce clinically significant changes in symptoms remain uncertain. Future research should aim to examine the trajectory of change across CBMT sessions, to identify whether a dose–response relationship exists, and if so, the optimal dose needed to have therapeutic effects and with what frequency of sessions. Future studies could then be designed using an evidence‐based definition of the number of sessions needed to qualify as a “completer.”

### 4.3 Clinical implications

This small study provides preliminary evidence that a multi‐session, CBMT intervention may be a helpful adjunct to treatment in a clinical sample of adolescents with eating disorders, and lay the foundation for larger, fully‐powered trials in representative samples. If this training is found to be effective in larger and more representative samples, it could be easily integrated into clinical practice due to several advantages. These include low cost, accessibility, no need for assistance from a therapist, and no side effects. However, before integrating the training into clinical practice, it is important to first explore the possible dose–response relationship between the training and key outcomes (e.g., bias change and clinical symptoms), and to assess whether any changes that are found are maintained over a follow‐up period. Furthermore, it is important to examine the baseline characteristics of participants who experience improvements in their symptoms following the training. This information would help to guide clinicians on whether to offer the training to all patients or to a sub‐group, such as those who display cognitive biases at baseline. Finally, it would be interesting to examine whether adapting the content of the interpretation bias modification modules further could improve social functioning, and whether adding content to the training to help with emotional processing of rejection may lead to changes in response to rejection, alongside changes interpretations of ambiguity.

## 5 CONCLUSION

This study demonstrated that a multi‐session online CBMT + TAU intervention effectively reduced negative interpretation biases and eating disorder psychopathology in adolescents with eating disorders to a greater extent than TAU only. However, the study provided no sufficient evidence that CBMT + TAU can reduce attention biases toward negative facial expressions, emotional response to criticism, anxiety or depression symptoms to a greater extent than TAU. As this is the first study to examine the effects of this training in adolescents with eating disorders, further studies should examine whether these findings are replicated, and empirically test the role of the training in producing clinical changes. It is also important to establish the stability of changes in interpretation bias and symptoms over time, and to further explore the impact of the training on emotional response to criticism and psychological distress. Finally, studies to identify the facilitators and barriers to engagement with CBMT in this patient group would be of benefit.

## AUTHOR CONTRIBUTIONS

**Katie Paige Rowlands:** Conceptualization; data curation; formal analysis; investigation; methodology; project administration; writing – original draft; writing – review and editing. **Taryn Beaty:** Data curation; project administration; writing – original draft; writing – review and editing. **Mima Simic:** Conceptualization; formal analysis; investigation; methodology; project administration; writing – original draft; writing – review and editing. **Ben Grafton:** Conceptualization; data curation; formal analysis; investigation; methodology; writing – original draft; writing – review and editing. **Colette Hirsch:** Conceptualization; methodology; writing – original draft; writing – review and editing. **Janet Treasure:** Conceptualization; formal analysis; investigation; methodology; project administration; supervision; writing – original draft; writing – review and editing. **Valentina Cardi:** Conceptualization; data curation; formal analysis; investigation; methodology; project administration; supervision; writing – original draft; writing – review and editing.

## CONFLICT OF INTEREST

Authors report no conflicts of interest.

## ETHICS STATEMENT

This study involved human participants and was reviewed and approved by London—Riverside Research Ethics Committee (REC Ref: 17/LO/1867). According to our ethically approved protocol, participants aged below 16 years of age provided assent to participate in this study. Parental consent was also obtained.

## Supporting information


**Item 1**. Supplementary description of attention bias assessment task
**Item 2**. Supplementary description of the study procedure
**Table S1**. Examples of positive, negative and catch trials in the ambiguous scenarios training task.
**Table S2**. Demographic and clinical characteristics of participants in the cognitive bias modification training + treatment as usual (CBMT + TAU) and TAU groups
**Table S3**. Cognitive biases, clinical measures and emotional response to criticism at baseline and post‐intervention in the cognitive bias modification training + treatment as usual (CBMT + TAU) and TAU groups

## Data Availability

The data supporting the findings of this study are available from the corresponding author upon reasonable request.
