# Supplementary material for: Cognitive bias modification training of attention and interpretation to reduce expectations of social rejection in adolescents with eating disorders: A small efficacy randomized controlled trial
Source: Int J Eat Disord. 2022 Sep 22;55(11):1506–20. doi: 10.1002/eat.23809 (PMC9825839; doi:10.1002/eat.23809)
Supplement: Supplementary file 1 — Item 1. Supplementary description of attention bias assessment task Item 2. Supplementary description of the study procedure Table S1. Examples of positive, negative and catch trials in the ambiguous scenarios training task. Table S2. Demographic and clinical characteristics of participants in the cognitive bias modification training + treatment as usual (CBMT + TAU) and TAU groups Table S3. Cognitive biases, clinical measures and emotional response to criticism at baseline and post‐intervention in the cognitive bias modification training + treatment as usual (CBMT + TAU) and TAU groups [file EAT-55-1506-s001.docx]

Supplementary Materials

**Item 1. Supplementary description of attention bias assessment task**

Subset A included face identities that were different from the face identities included in Subset B. Participants were randomised to either complete the assessment using faces from Subset A or Subset B. Three face images were selected for each individual, including a facial expression of criticism, a happy smile, and a neutral facial expression. For each face pair, an emotional facial expression (i.e. either the critical or happy expression) was presented next to a neutral facial expression, of the same individual. Following a fixation cross ( + ) presented for 500ms, each face pair was presented for 500ms, until the participant responded. The inter-trial interval duration was 500ms. The task included 16 practice trials (using separate faces not used in the real trials) and 128 assessment trials, across two blocks of 64 trials. There were an equal number of trials on which a male vs. female face were presented, an equal number of trials on which the emotional face appeared on the left vs. right-hand side of the screen, and an equal number of trials on which the probe appeared in the same vs. opposite location of the emotional face. At baseline, each face identity was presented once, before being presented again. The first time the face was shown it was either presented showing a rejecting frown or a happy smiling expression, and the second time it was presented showing the opposite expression. At end of intervention, each face identity was presented once and the expression was either critical or happy, selected at random.

**Item 3. Supplementary description of ambiguous scenarios training task**

In all trials, following the presentation of each scenario, participants were asked a ‘comprehension question’ which they had to answer “yes” or “no” to. In modification trials, the answer to this comprehension question was followed by feedback (for 1000ms) indicating whether the answer was correct or by a sound indicating that the answer was incorrect, followed by the correct answer highlighted in red text. On catch trials, no feedback was provided. The catch trials were presented at random points in between the modification trials. The wording of scenarios and comprehension questions were balanced, so that the “correct” answer was “yes” or “no” with equal contingencies, which could reinforce either a positive or negative interpretation. See Figure 1 for an example of each trial type. Participants were encouraged to complete a total of nine CBM-I training sessions.

**Item 4. Supplementary description of the study procedure**

The study was advertised using a flyer and brief study summary to patients by psychology staff at two child and adolescent eating disorder services, through a carer group mailing list, and via online advertisements (e.g. Beat, Twitter, Callforparticipants). Patients who expressed an interest in the study were asked some basic screening questions via email to confirm their eligibility. Patients and parents for those under the age of 16 were provided with an information sheet and had the opportunity to ask questions about the study via email or over the telephone. Informed consent was obtained for all participants and from parents of patients under the age of 16. Patients who consented to take part were provided with a unique anonymous study ID number, and were then sent a link to the online psychological testing platform “Inquisit” (<https://www.millisecond.com/products/inquisit6/weboverview.aspx>) along with instructions to complete the baseline measures which took approximately 30 minutes. Participants were randomised using random numbers generated on an excel spreadsheet. Participants were randomly allocated to one of the two study conditions, CBMT+TAU or TAU only, based on 50% probability of being allocated to each condition. The CBMT+TAU group were then sent the nine links to the CBMT sessions over the first four weeks of their participation. All sessions started with the attention bias modification paradigm, followed by the interpretation bias modification paradigm. Each training session lasted for approximately 40 minutes. Participants were encouraged to complete 2-3 sessions per week and to take regular breaks during the tasks and in between sessions. All participants were sent weekly email reminders to complete the sessions. Participants allocated to both groups were then sent a link to the end of intervention questionnaires and computerised tasks. All participants received £30 reimbursement for their contribution to the data collection.

**Supplementary Table 1**

Examples of positive, negative and catch trials in the Ambiguous Scenarios Training Task.

| Trial type | Scenario | Comprehension question | Feedback |
| --- | --- | --- | --- |
| Positive trial | You ask a member of your family to have a look at some school work you are really proud of. When they read the first paragraph, they look...**interested.”** | Do your family pay attention to your work?" | Yes = correct  No = incorrect |
| Negative trial | "You wait at a busy platform for a bus. As you get onto the bus you walk towards the back and stop. A business man next to you asks if you would mind moving down. In doing so, he looks…**annoyed."** | Does the man have a negative attitude towards you?" | Yes = correct  No = incorrect |
| Catch trial | "You are introduced to someone at a party and receive a follow request from them on Instagram the following day. You accept their request and send them a message to say hello. Later that week you realise you haven’t received a reply and try to find their account online. They seem to have**…disappeared."** | Has this person taken a break from social media?" | No feedback |

| **Supplementary Table 2** | | | | | |
| --- | --- | --- | --- | --- | --- |
|  |  |  |  |  |  |
| *Demographic and Clinical Characteristics of Participants in the Cognitive Bias Modification Training + Treatment as Usual (CBMT + TAU) and TAU Groups* | | | | |  |
|  |  |  |  |  |  |
|  |  |  |  |  |  |
| Baseline characteristics | All (N = 67) | CBMT + TAU (n = 37) | TAU (n = 30) | Test statistic, significance | Effect size |
|  | *N (%) or M (SD)* | | |  |  |
| Age | 16.06 (1.70) | 16.54 (1.50) | 15.47 (1.78) | *t*(65)= 2.68, *p* = .275 | *d* = .65 |
| Gender |  |  |  | P = | φ = .111 |
| Girls | 66 | 36 | 30 |  |  |
| Boys | 1 | 1 | 0 |  |  |
| Ethnicity |  |  |  | *p =* .085 | φ = .240 |
| White British | 64 | 37 | 27 |  |  |
| Asian | 2 | 0 | 2 |  |  |
| Other | 1 | 0 | 1 |  |  |
| Eating disorder diagnosis |  |  |  | *p* = .260 | φ = ..247 |
| Anorexia nervosa | 63 | 35 | 28 |  |  |
| Bulimia nervosa | 2 | 2 | 0 |  |  |
|  | 1 | 0 | 1 |  |  |
| EDNOS | 1 | 0 | 1 |  |  |
| Duration of eating disorder (months) | 19.45 (21.49) | 25.92 (23.84) | 11.47 (15.04) | *t*(65)= 2.885, *p* = .005 | *d* = .74 |
| Comorbid diagnoses | 29 | 19 | 10 | *p* = *.*215 | φ = .181 |
| Depression | 22 | 17 | 5 |  |  |
| Bipolar disorder |  |  |  |  |  |
| Anxiety disorder | 17 | 13 | 4 |  |  |
| ASD | 3 | 3 | 0 |  |  |
| OCD | 2 | 2 | 0 |  |  |
| Multiple comorbidities | 15 | 13 | 2 |  |  |
| Treatment |  |  |  | *p* = .528 | φ = .152 |
| Outpatient | 60 | 33 | 27 |  |  |
| Day care | 6 | 4 | 2 |  |  |
| Inpatient | 1 | 0 | 1 |  |  |
| Psychiatric medication | 29 | 14 | 15 | *p*  = .335 | φ = -.122 |
| Weight for height percentile | 22.95 (21.97) | 22.56 (23.00) | 23.41 (21.11) | *t*(57)= -.146, *p* = .885 | *d* = .04 |
| IPSM Interpersonal awareness | 24.81 (3.13) | 25.08 (2.98) | 24.47 (3.34) | *t*(65)= .796, *p* = .429 | *d* = .19 |
| IPSM Need for approval | 25.90 (2.77) | 26.22 (2.57) | 25.50 (2.99) | *t*(65)= 1.053, *p* = .296 | *d* = .26 |
| IPSM Separation anxiety | 24.61 (4.36) | 25.00 (4.37) | 24.13 (4.38) | *t*(65)= .807, *p* = .423 | *d* = .20 |
| IPSM Timidity | 23.69 (4.34) | 23.95 (3.96) | 23.37 (4.81) | *t*(65)= .-541, *p* = .591 | *d* = .13 |
| IPSM Fragile inner self | 13.81 (3.53) | 14.22 (3.22) | 13.30 (3.87) | *t*(65)= 1.059, *p* = .294 | *d* = .26 |
| Interpersonal sensitivity total | 112.81 (12.54) | 114.46 (11.71) | 110.77 (13.42) | *t*(65)= .181, *p* = .234 | *d* = .29 |
| EDE-Q Eating restraint | 3.54 (1.71) | 3.73 (1.70) | 3.30 (1.71) | *t*(65)= -.1.025, *p* = .309 | *d* = .25 |
| EDE-Q Eating concerns | 3.33 (1.39) | 3.36 (1.40) | 3.30 (1.39) | *t*(65)= .165, *p* = .869 | *d* = .04 |
| EDE-Q Weight concerns | 4.10 (1.55) | 4.10 (1.46) | 4.09 (1.68) | *t*(65)= -.140, *p* = .889 | *d* = .01 |
| EDE-Q Shape concerns | 4.72 (1.34) | 4.70 (1.18) | 4.75 (1.53) | *t*(65)= .010, *p* = .992 | *d* = .04 |
| EDE-Q total | 3.92 (1.36) | 3.97 (1.31) | 3.86 (1.44) | *t*(65)= -.330, *p* = .743 | *d* = .08 |
| RCADS-25 Depression | 71.40 (16.79) | 72.61 (16.77) | 69.91 (16.99) | *t*(65)= .651, *p* = .517 | *d* = .16 |
| RCADS-25 Anxiety | 63.67 (13.85) | 65.82 (14.09) | 61.03 (13.30) | *t*(65)= 1.416, *p* = .162 | *d* = .06 |
| RCADS-25 Total | 69.29 (16.03) | 71.39 (16.41) | 66.70 (15.42) | *t*(65)= .784, *p* = .237 | *d* = .29 |
| *Note.* This table displays demographic and clinical characteristics collected from all participants at baseline. The table presents data for the sample as a whole (N = 67) and for the two study groups including the Cognitive Bias Modification Training + Treatment as Usual (CBMT + TAU) and the TAU groups. Abbreviations include ARFID (Avoidant Restrictive Food Intake Disorder), EDNOS (Eating Disorder Not Otherwise Specified), ASD (Autism Spectrum Disorder), OCD (Obsessive Compulsive Disorder), IPSM (Interpersonal Sensitivity Measure), EDE-Q (Eating Disorder Examination - Questionnaire) and RCADS-25 (Revised Child Anxiety and Depression Scale - Short Form). Data presented as means (*M*), standard deviations (*SD*) or counts with percentages, test statistics, and significance values. Φ = Phi. d = Cohen's d measure of effect size. | | | | | |

**Supplementary Table 3**

*Cognitive Biases, Clinical Measures and Emotional Response to Criticism at Baseline and Post-intervention in the Cognitive Bias Modification Training + Treatment as Usual (CBMT + TAU) and TAU Groups*

|  | Baseline M (SD) | Post-intervention M (SD) | Mean difference M [95% CI] | Baseline M (SD) | Post-intervention M (SD) | Mean difference M [95% CI] | Test Statistics |
| --- | --- | --- | --- | --- | --- | --- | --- |
|  | CBMT + TAU n = 24 | | |  | TAU n = 26 | |  |
| Total negative completions | 26.17 (12.92) | 17.54 (12.56) | -8.63 -13.64 to -3.61] | 28.27 (14.68) | 23.58 (10.57) | -4.69 [-10.33 to .94] | Time F(1, 48) = 13.104, p = .001*, ηp2 = .214 |
|  |  |  |  |  |  |  | Group F(1, 48) = 1.711, p = .197, ηp2 = ..034 |
|  |  |  |  |  |  |  | Time x Group F(1, 48) = .1.143, p = .290, ηp2 = .023 |
| First negative completions | 6.00 (1.29) | 4.25 (1.70) | -1.75 [-2.57 to -.93] | 6.54 (2.60) | 6.12 (1.84) | -.42 [-1.39 to .547] | Time F(1, 48) = 12.254, p = .001*, ηp2 = .203 |
|  |  |  |  |  |  |  | Group F(1, 48) = 7.146, p = .010*, ηp2 = .130 |
|  |  |  |  |  |  |  | Time x Group F(1, 48) = 4.569, p = .038*, ηp2 = .087 |
| Best negative completions | 5.79 (1.93) | 3.71 (2.16) | -2.08 [-3.01 to 1.15] | 5.50 (3.02) | 5.73 (2.13) | .23 [-.78 to 1.24] | Time F(1, 45) = ..064, p = .802, ηp2 = .001 |
|  |  |  |  |  |  |  | Group F(1, 45) = 2.661, p = .110, ηp2 = .056 |
|  |  |  |  |  |  |  | Time x Group F(1, 45) = 7.388, p = .009*, ηp2 = .141 |
|  |  |  |  |  |  |  | Time x Depression F(1, 45) = .106, p = .746, ηp2 = .002 |
|  |  |  |  |  |  |  | Time x Anxiety F(1, 45) = .172, p = .680, ηp2 = .004 |
|  |  |  |  |  |  |  | Time x Duration of eating disorder F(1, 45) = .665, p = .419, ηp2 = .051 |
| Attention bias to critical faces | -7.25 (103.69) | -55.54 (473.05) | -44.62[-245.07 to 155.83] | -19.92 (81.97) | -42.58 (164.56) | -6.64 [79.84 to 66.56] | Time F(1, 47) = .455, p = .503, ηp2 = .010 |
|  |  |  |  |  |  |  | Group F(1, 47) = .000, p = 998, ηp2 = .000 |
|  |  |  |  |  |  |  | Time x Group F(1, 47) = .059, p = .809, ηp2 = .001 |
| EDE-Q Total | 3.65 (1.40) | 3.16 (1.41) | -.48 [-.84 to -.13] | 3.79 (1.46) | 3.64 (1.47) | -.15 [-.33 to .04] | Time F(1, 51) = 10.974, p = .002, ηp2 = .177 |
|  |  |  |  |  |  |  | Group F(1, 51) = .667, p = .418, ηp2 = .013 |
|  |  |  |  |  |  |  | Time x Group F(1, 51) = 3.132, p = .083, ηp2 = .058 |
| RCADS-25 Anxiety | 67.03 (16.50) | 64.37 (17.00) | -2.66 [-7.72 to 2.41] | 60.92 (13.73) | 57.15 (15.33) | -3.76 [-8.5 to .97] | Time F(1, 51) = 3.640, p = .062, ηp2 =.067 |
|  |  |  |  |  |  |  | Group F(1, 51) = 2.833, p = .098, ηp2 = .053 |
|  |  |  |  |  |  |  | Time x Group F(1, 51) = .109, p = .743, ηp2 = .002 |
| RCADS-25 Depression | 69.74 (19.49) | 66.93 (17.08) | -2.88 [-8.13 to 2.37] | 69.38 (17.29) | 66.12 (17.33) | -3.26 [-8.45 to 1.92] | Time F(1, 48) = 2.558, p = .116, ηp2 = .051 |
|  |  |  |  |  |  |  | Group F(1, 48) = .016, p = .901, ηp2 = .000 |
|  |  |  |  |  |  |  | Time x Group F(1, 48) = .014, p = .907, ηp2 = .000 |
| RCADS-25 Total | 70.55 (18.61) | 67.67 (16.26) | -3.15 [-8.92 to 2.62] | 69.38 (17.29) | 66.12 (17.33) | 4.03 [-1.25 to 9.31] | Time F(1, 51) = 2.915, p = .094, ηp2 = .054 |
|  |  |  |  |  |  |  | Group F(1, 51) = .094, p = .760, ηp2 = .002 |
|  |  |  |  |  |  |  | Time x Group F(1, 51) = .011, p = .915, ηp2 = .000 |
| Emotional response to criticism | | 11.88 (5.38) |  |  | 14.04 (4.04) |  | *U* = 176, p = .262 |

*Note.* This table presents data for the main study variables collected from participants at baseline and post-intervention. Abbreviations include EDE-Q (Eating Disorder Examination Questionnaire), and RCADS-25 (Revised Children’s Anxiety and Depression Scale – Short Form). Analyses of negative "best" completions and EDE-Q total include depression, anxiety and eating disorder duration as covariates. Emotional response to criticism = combined score on visual analogue scale after presentation of video sets 1 and 2. Data presented as means (*M*), standard deviations (*SD*), mean difference, 95% confidence intervals, test statistics, significance values, and ηp2 = Partial eta squared (effect sizes).

**Supplementary Table 4. Self-report feedback from participants in the CBMT + TAU Group**

| Participant | Feedback | Suggestions to support participant engagement | Suggestions to improve the programme |
| --- | --- | --- | --- |
| 1 | “Some of the tasks were strange”  “Helped me to think differently” | “None - the support was great” | “Not sure” |
| 2 | “It made me question my social interactions and engage in challenges rather than avoid them”  “Software kept crashing”  “It has made me more aware of how I interpret threat/negative signals in neutral situations and always expect the worst” | “Nothing more!” | “Maybe shorter tasks” |
| 3 | “Met my expectations by being personal and helpful to me”  “Realised that I was not thinking about social interactions very flexibly and helped me to think about how I could manage them in a more relaxed way. I have more confidence with approaching people now” | “Maybe more information about the tasks and their aims” | “Less technical glitches”  “Technical difficulties”  “Limitations due to COVID” |
| 4 |  |  | “Less attention tasks” |
| 5 | “Could do it on my own” |  |  |
| 6 | “Simpler than expecting”  “Some had low sound”  “More open-minded” |  | “Pre-recorded video to explain” |
| 7 | “More repetitive than expected”  “Different volumes of audio clips”  “Didn't feel an impact” | “None” | “Consistent volume” |
| 8 | “At first took a while”  “Got me thinking” | “Already good support” | “None, it was simple” |
| 9 |  | “Include what is the point, what they were achieving” | “Less yes + no questions” |
| 10 | “It helped me to identify the final things I need to work on in the final bits of my recovery. It made me realise things that I still needed to work on”  “the attention bias tasks at the start were quite monotonous and I personally couldn't see the benefit”  “The tasks where you would listen to the clips then pressed yes or no really helped me to deal with social situations. It reinforced things I'd been told in therapy and reminded me not to think too much into things. It helped me identify what is just the anxiety talking and what is actually logical, e.g. if my classmates are smiling while I'm doing a presentation, I must not let anxiety get a handle and must realise that they are probably just smiling because they're proud or think the presentation is well done. On the other hand, it still allowed me to know when people are being unkind or are 'toxic' for example if they're laughing if I fall or they say something rude to me. This reinforced my self-worth, made me realise that I should still recognise when people are bad, unkind, mean etc. It really helped me and I could put this into social situations. The main point I took from it was to not just assume that someone's impression of me or actions towards me are negative - to think logically not letting negativity or anxiety take over.” | “I think they did everything they could. Nothing else I needed really” | “Maybe we could only do the attention bias task once a week instead of 3 times.” |
| 11 | “It did as I’ve done similar”  “Tech problems e.g. freezing” | “More different tasks” | “Less repetitive” |
| 12 | “Didn’t understand the line one” | “Info on what interventions do”  “Shorter questionnaires” |  |
| 13 | “It was an enjoyable study” | “Shorter tasks” |  |
| 14 | “Difficulties maintaining focus”  “Helped change perspectives” | “Task reminders” |  |
| 15 |  |  | “Less questions” |
| 16 | “The questionnaires really made me stop and think about how bad my habits are and I realised how much they were affecting my life”  “Sometimes the words were cut off the screen and sentences were harder to understand” | “I found the support to be good throughout, researchers were understanding when I was late with responses etc. the team was approachable and empathetic” |  |
| 17 | “The program helped me practice what I learned with my behaviour therapist, coaching, and/or groups. Some things were completely new to what I'd done but some of it was similar and really helped especially in recognising when I'm making assumptions that are influenced by my social anxiety and thus are not rational or true”  “at times it didn't work . quite repetitive at times”  “Overall I thought it helped and I saw a difference however I think it could get slightly too repetitive and longwinded.”  “Not necessarily relevant for all of them but useful to think about how I would respond or react to those scenarios or situations.”  “The horizontal/vertical line task was fun. I would try to see how little mistakes I could make“ |  |  |
